# The Translated Dowling Polynomials and Numbers

**DOI:** 10.1155/2014/678408

**Published:** 2014-12-22

**Authors:** Mahid M. Mangontarum, Amila P. Macodi-Ringia, Normalah S. Abdulcarim

**Affiliations:** Department of Mathematics, Mindanao State University, Main Campus, 9700 Marawi City, Philippines

## Abstract

More properties for the translated Whitney numbers of the second kind such as horizontal generating function, explicit formula, and exponential generating function are proposed. Using the translated Whitney numbers of the second kind, we will define the translated Dowling polynomials and numbers. Basic properties such as exponential generating functions and explicit formula for the translated Dowling polynomials and numbers are obtained. Convexity, integral representation, and other interesting identities are also investigated and presented. We show that the properties obtained are generalizations of some of the known results involving the classical Bell polynomials and numbers. Lastly, we established the Hankel transform of the translated Dowling numbers.

## 1. Introduction

In 1996, the classical* Whitney numbers of the second kind W*_*m*_(*n*, *k*)* of Dowling Lattices* was introduced by Benoumhani [1]. *W*_*m*_(*n*, *k*) satisfy the recurrence relation:

(1) $$\begin{array}{c} W_{m}\left(n,k\right)=W_{m}\left(n-1,k-1\right)+\left(1+mk\right)W_{m}\left(n-1,k\right). \end{array}$$

Other fundamental properties of these numbers were already established by Benoumhani in [1, 2]. The numbers *W*_*m*_(*n*, *k*) can be shown to be a kind of generalization of the famous* Stirling numbers of the Second kind S*(*n*, *k*) when the parameter *m* equals to 1. That is,

(2) $$\begin{array}{c} W_{1}\left(n,k\right)=S\left(n,k\right). \end{array}$$

Recently, a translated version of the Whitney numbers of the second kind was introduced by Belbachir and Bousbaa [3] which they named* translated Whitney numbers of the second kind*, denoted by ${\tilde{W}}_{\left(\alpha \right)}(n,k)$. ${\tilde{W}}_{\left(\alpha \right)}(n,k)$ actually counts the number of partitions of a set with *n* elements into *k* subsets such that the elements of each subset can mutate in *α* ways, except the dominant one. To compute the first few values of these numbers, the following recurrence relation was obtained in [3]:

(3) $$\begin{array}{c} {\tilde{W}}_{\left(\alpha \right)}\left(n,k\right)={\tilde{W}}_{\left(\alpha \right)}\left(n-1,k-1\right)+k\alpha {\tilde{W}}_{\left(\alpha \right)}\left(n-1,k\right). \end{array}$$

The classical Stirling numbers of the second kind can also be obtained from these numbers when *α* = 1. On the otherhand, the classical* Dowling numbers D*_*m*_(*n*) are defined to be the sum of  *W*_*m*_(*n*, *k*). That is,

(4) $$\begin{array}{c} D_{m}\left(n\right)=\overset{n}{\underset{k=0}{\sum }}W_{m}\left(n,k\right) \end{array}$$

and can be computed using the explicit formula:

(5) $$\begin{array}{c} D_{m}\left(n\right)=\frac{1}{e^{1/m}}\underset{k\geq 0}{\sum }\frac{{\left(mk+1\right)}^{n}}{m^{k}k!}. \end{array}$$

*D*_*m*_(*n*) is known to be a generalization of the classical* Bell numbers* which is the sum of the Stirling numbers of the second kind *S*(*n*, *k*). In this paper, we will define the* translated Dowling* numbers as the sum of ${\tilde{W}}_{\left(\alpha \right)}(n,k)$. The content of this paper is summarized as follows. In Section 2, we will introduce some basic properties for the numbers ${\tilde{W}}_{\left(\alpha \right)}(n,k)$. In Section 3, we will define the translated Dowling polynomials and numbers and derive some of their basic properties. In Section 4, we investigate convexity and integral representation of the translated Dowling polynomials and numbers. In Section 5, more properties of translated Dowling polynomials and numbers are presented, and in Section 6, we obtain the Hankel transform of the translated Dowling numbers.

## 2. Some Properties of ${\tilde{W}}_{\left(\alpha \right)}(n,\;k)$

Interesting properties of ${\tilde{W}}_{\left(\alpha \right)}(n,k)$ can also be obtained parallel to those done in [1]. For instance, by induction on *n*, the following horizontal generating function can easily be obtained through the aid of the recurrence relation in (3).

**Proposition 1 .**

The translated Whitney numbers of the second kind satisfy the following horizontal generating function:

(6) $$\begin{array}{c} t^{n}=\overset{n}{\underset{k=0}{\sum }}{\tilde{W}}_{\left(\alpha \right)}\left(n,k\right){\left(t\;|\;\alpha \right)}_{k}, \end{array}$$

where (*t*∣*α*)_*k*_ = ∏_*i*=0_^*k*−1^(*t* − *iα*) is the generalized factorial of *t* of increment *α*.

Also, note that (6) can be written as

(7) $$\begin{array}{c} t^{n}=\overset{n}{\underset{k=0}{\sum }}{\tilde{W}}_{\left(\alpha \right)}\left(n,k\right)\alpha^{k}{\left(\frac{t}{\alpha }\right)}_{k}, \end{array}$$

where (*t*/*α*)_*k*_ is the falling factorial of *t*/*α* of order *k*. By replacing *t* with *kα*, we have

(8) αkn=∑j=0nW~(α)(n,j)αjkj,=∑j=0nkjW~(α)(n,j)αjkjkj.

Finally, applying the binomial inversion formula (see [4])

(9) $$\begin{array}{c} f_{k}=\overset{k}{\underset{j=0}{\sum }}\left(\begin{array}{c} k\\ j \end{array}\right)g_{j}⟺g_{k}=\overset{k}{\underset{j=0}{\sum }}{\left(-1\right)}^{k-j}\left(\begin{array}{c} k\\ j \end{array}\right)f_{j} \end{array}$$

gives us the following explicit formula.

**Proposition 2 .**

The translated Whitney numbers of the second kind can be expressed as

(10) $$\begin{array}{c} {\tilde{W}}_{\left(\alpha \right)}\left(n,k\right)=\frac{1}{\alpha^{k}k!}\overset{k}{\underset{j=0}{\sum }}{\left(-1\right)}^{k-j}\left(\begin{array}{c} k\\ j \end{array}\right){\left(\alpha j\right)}^{n}. \end{array}$$

Note that when *α* = 1 in (10), we have

(11) W~1n,k=1k!∑j=0k−1k−jkjjn=S(n,k),

which is the known explicit formula of the Stirling numbers of the second kind. Furthermore, we have the following exponential generating function.

**Proposition 3 .**

The numbers ${\tilde{W}}_{\left(\alpha \right)}(n,k)$ satisfy

(12) $$\begin{array}{c} \underset{n\geq k}{\sum }{\tilde{W}}_{\left(\alpha \right)}\left(n,k\right)\frac{z^{n}}{n!}=\frac{1}{k!}{\left(\frac{e^{\alpha k}-1}{\alpha }\right)}^{k}. \end{array}$$

**Proof**

Multiplying both sides of (8) by *z* ^*n*^/*n*! and summing over *n*, gives us

(13) ∑n≥0αtnznn!=∑n≥0 ∑k=0nW~αn,kαktkznn!=∑k≥0αk∑n≥kW~(α)(n,k)znn!tk.

Now, note that

(14) ∑n≥0αtnznn!=1+eαz−1t=∑k=0ttkeαz−1k=∑k=0teαz−1kk!tk.

The proof is completed by comparing the coefficients of (*t*)_*k*_ in (13) and (14).

In 2010, Mező [5], introduced the *r*-Whitney numbers of the second kind *W*_*m*,*r*_(*n*, *k*) as coefficients in the expansion of

(15) $$\begin{array}{c} {\left(mx+r\right)}^{n}=\overset{n}{\underset{k=0}{\sum }}m^{k}W_{m,r}\left(n,k\right){\left(x\right)}_{k}. \end{array}$$

These numbers actually are equivalent to the (*r*, *β*)-Stirling numbers ${\langle \begin{array}{c} n\\ k \end{array}\rangle }_{r,\beta }$ defined by Corcino et al. [6]. That is,

(16) $$\begin{array}{c} W_{\beta ,r}\left(n,k\right)={\langle \begin{array}{c} n\\ k \end{array}\rangle }_{r,\beta }. \end{array}$$

Moreover, we have

(17) $$\begin{array}{c} W_{1,0}\left(n,k\right)=S\left(n,k\right),\\ W_{1,r}\left(n,k\right)={\left\{\begin{array}{c} n+r\\ k+r \end{array}\right\}}_{r},\\ W_{m,0}\left(n,k\right)=W_{m}\left(n,k\right),\\ W_{\alpha ,0}\left(n,k\right)={\tilde{W}}_{\left(\alpha \right)}\left(n,k\right), \end{array}$$

where ${\left\{\begin{array}{c} n+r\\ k+r \end{array}\right\}}_{r}$ is the *r*-Stirling numbers of the second kind by Broder [7]. This means that the identities (6), (10), and (12) for the numbers ${\tilde{W}}_{\left(\alpha \right)}(n,k)$ appear to be special cases of *W*_*m*,*r*_(*n*, *k*) (see [5, 8]).

## 3. Translated Dowling Polynomials and Numbers

The well-known* Bell polynomials B*_*n*_(*x*) is defined by the sum

(18) $$\begin{array}{c} B_{n}\left(x\right)=\overset{n}{\underset{k=0}{\sum }}S\left(n,k\right)x^{k} \end{array}$$

which consequently yields the Bell numbers *B*_*n*_ when *x* = 1. In line with this, we may define the translated Dowling polynomials as follows.

**Definition 4 .**

For nonnegative integers *n*, *k*, and *α*, the translated Dowling polynomials are defined as

(19) $$\begin{array}{c} {\tilde{D}}_{\left(\alpha \right)}\left(n;x\right)=\overset{n}{\underset{k=0}{\sum }}{\tilde{W}}_{\left(\alpha \right)}\left(n,k\right)x^{k}. \end{array}$$

When *x* = 1,

(20) $$\begin{array}{c} {\tilde{D}}_{\left(\alpha \right)}\left(n;1\right)={\tilde{D}}_{\left(\alpha \right)}\left(n\right)=\overset{n}{\underset{k=0}{\sum }}{\tilde{W}}_{\left(\alpha \right)}\left(n,k\right) \end{array}$$

and is called the translated Dowling numbers.

Now, from (19) and (12),

(21) ∑n≥0D~αn;xznn!=∑n≥0 ‍∑k=0nW~(α)(n,k)znn!xk=∑k≥0eαz−1kk!αkxk=ex(eαz−1)/α.

Hence, we have the following theorem.

**Theorem 5 .**

The following exponential generating functions hold:

(22) $$\begin{array}{c} \underset{n\geq 0}{\sum }{\tilde{D}}_{\left(\alpha \right)}\left(n;x\right)\frac{z^{n}}{n!}=\exp\left\{\frac{x\left(e^{z\alpha }-1\right)}{\alpha }\right\}; \end{array}$$

(23) $$\begin{array}{c} \underset{n\geq 0}{\sum }{\tilde{D}}_{\left(\alpha \right)}\left(n\right)\frac{z^{n}}{n!}=\exp\left\{\frac{e^{z\alpha }-1}{\alpha }\right\}. \end{array}$$

**Remark 6 .**

When *α* = 1 in (22) and (23), we have

(24) $$\begin{array}{c} \underset{n\geq 0}{\sum }{\tilde{D}}_{\left(1\right)}\left(n;x\right)\frac{z^{n}}{n!}=e^{x\left(e^{z}-1\right)};\\ \underset{n\geq 0}{\sum }{\tilde{D}}_{\left(1\right)}\left(n\right)\frac{z^{n}}{n!}=e^{e^{z}-1}, \end{array}$$

which are the exponential generating functions of the classical Bell polynomials and numbers, respectively.

Since ${\tilde{W}}_{\left(\alpha \right)}(n,k)$ represents the number of partitions of a set with *n* elements into *k* subsets such that the elements of each subset can mutate in *α* ways, except the dominant one, then ${\tilde{D}}_{\left(\alpha \right)}(n)$ is the number of partitions of a set with *n* elements such that the elements of each subset can mutate in *α* ways, except the dominant one. The following theorem contains an explicit form for the polynomials ${\tilde{D}}_{\left(\alpha \right)}(n;x)$ and numbers ${\tilde{D}}_{\left(\alpha \right)}(n)$.

**Theorem 7 .**

The following explicit formula holds:

(25) $$\begin{array}{c} {\tilde{D}}_{\left(\alpha \right)}\left(n;x\right)={\left(\frac{1}{e}\right)}^{x/\alpha }\underset{i\geq 0}{\sum }\frac{{\left(i\alpha \right)}^{n}}{i!}{\left(\frac{x}{\alpha }\right)}^{i}; \end{array}$$

(26) $$\begin{array}{c} {\tilde{D}}_{\left(\alpha \right)}\left(n\right)={\left(\frac{1}{e}\right)}^{1/\alpha }\underset{i\geq 0}{\sum }\frac{{\left(i\alpha \right)}^{n}}{i!\alpha^{i}}. \end{array}$$

**Proof**

Combining the explicit formula in (10) with (19) yields

(27) $$\begin{array}{c} {\tilde{D}}_{\left(\alpha \right)}\left(n;x\right)=\underset{j\geq 0}{\sum }\;\underset{k\geq j}{\sum }\frac{{\left(-1\right)}^{j}\left(\begin{array}{c} k\\ j \end{array}\right){\left(k-j\right)}^{n}}{k!}\alpha^{n-k}x^{k}. \end{array}$$

Reindexing the sums and by further simplification,

(28) D~αn;x=∑j≥0−xjαjj!∑i≥0inxiαii!αn=e−x/α∑i≥0iαni!xαi.

Equation (26) is obtained by letting *x* = 1.

**Remark 8 .**

When *α* = 1 in (25) and (26), we have

(29) $$\begin{array}{c} {\tilde{D}}_{\left(1\right)}\left(n;x\right)=\frac{1}{e^{x}}\underset{i\geq 0}{\sum }\frac{i^{n}}{i!}x^{i}=B_{n}\left(x\right);\\ {\tilde{D}}_{\left(1\right)}\left(n\right)=\frac{1}{e^{x}}\underset{i\geq 0}{\sum }\frac{i^{n}}{i!}=B_{n}, \end{array}$$

which are the known Dobinski identities.

To close this section, we will cite the *r*-Dowling polynomials *D*_*m*,*r*_(*n*, *x*) of Cheon and Jung [8] defined by

(30) $$\begin{array}{c} D_{m,r}\left(n,x\right)=\overset{n}{\underset{k=0}{\sum }}W_{m,r}\left(n,k\right)x^{k}. \end{array}$$

Properties of *D*_*m*,*r*_(*n*, *x*) were already established in [8] and were further studied by Rahmani [9]. We note that the polynomials *D*_*m*,*r*_(*n*, *x*) coincide with the (*r*, *β*)-Bell polynomials *G*_*n*,*β*,*r*_(*x*) of R. B. Corcino and C. B. Corcino [10]. That is, *D*_*β*,*r*_(*n*, *x*) = *G*_*n*,*β*,*r*_(*x*). Moreover,

(31) $$\begin{array}{c} D_{1,0}\left(n,x\right)=B_{n}\left(x\right),\;\;D_{1,r}\left(n,x\right)=B_{n,r}\left(x\right),\\ D_{\alpha ,0}\left(x\right)={\tilde{D}}_{\left(\alpha \right)}\left(n;x\right), \end{array}$$

where *B*_*n*,*r*_(*x*) is the *r*-Bell polynomials in [11].

## 4. Convexity and Integral Representation

A real sequence *v*_*k*_, *k* = 0,1, 2,… is called* convex* [4] on an interval [*a*, *b*], where [*a*, *b*] contains at least 3 consecutive integers, if

(32) $$\begin{array}{c} v_{k}\leq \frac{1}{2}\left(v_{k-1}+v_{k+1}\right),\;k\in \left[a+1,b-1\right]. \end{array}$$

We will refer to (32) as* convexity property*. Convexity, among others, is an example of interesting global behaviours of combinatorial sequences of integers. The following theorem shows that the polynomials ${\tilde{D}}_{\left(\alpha \right)}(n,x)$ obey the convexity property.

**Theorem 9 .**

Let *x* > 0 and *α* ≥ 0. Then

(33) $$\begin{array}{c} {\tilde{D}}_{\left(\alpha \right)}\left(n+1;x\right)\leq \frac{1}{2}\left[{\tilde{D}}_{\left(\alpha \right)}\left(n;x\right)+{\tilde{D}}_{\left(\alpha \right)}\left(n+2;x\right)\right] \end{array}$$

for *n* = 1,2, 3,….

**Proof**

Since *αk* ≥ 0, then

(34) 0≤1−αk20≤1−2(αk)+αk22αk≤1+αk22αkn+1≤αkn+αkn+2αkn+1≤12αkn+αkn+2.

Multiplying both sides by (*x*/*α*)^*k*^(1/*k*!) and summing over *i* yields

(35) ∑i≥0αkn+1i!xαi ≤12∑i≥0αkni!xαi+∑i≥0αkn+2i!xαi.

Finally, multiplying both sides by *e* ^−*x*/*α*^ and using (25) completes the proof.

The following beautiful integral representation of the Bell numbers *B*_*n*_ was first obtained by Cesàro [12]:

(36) Bn=2n!πeIm⁡∫0πeeeiθsin(nθ)dθ.

This expression was generalized by Mező [11] using a kind of generalization of the classical Bell numbers called *r*-Bell numbers *B*_*n*,*r*_. Equation (36) and Mező's identity appears to be special cases of the integral representation of the (*r*, *β*)-Bell polynomials *G*_*n*,*β*,*r*_(*x*) by R. B. Corcino and C. B. Corcino [10]. That is *G*_*n*,1,*r*_(1) = *B*_*n*,*r*_ and *G*_*n*,1,0_(1) = *B*_*n*_, respectively. The next theorem gives an integral representation for the translated Dowling polynomials.

**Theorem 10 .**

The translated Dowling polynomials have the following integral representation:

(37) D~(α)(n;x)=2n!πex/αIm⁡∫0πexp⁡xαeαeiθsin(nθ)dθ,

where $i=\sqrt{-1}$.

**Proof**

From [13], we have the following integral identity:

(38) Im⁡∫0πejeiθsin(nθ)dθ=π2jnn!.

Hence, combining this with the explicit formula in (10) yields

(39) π21n!W~αn,k =1αkk!∑j=0k−1k−jkjπ2αjnn! =1αkk!∑j=0k−1k−jkj     iiii×Im⁡∫0πeαjeiθsin⁡(nθ)dθ =1αkk!  ×Im⁡∫0π∑j=0k−1k−jkjeαeiθjsin⁡nθdθ =Im⁡∫0πeαeiθ−1kαkk!sin(nθ)dθ.

Furthermore, we have

(40) ∑k≥0W~αn,kxk =2n!πIm⁡∫0π∑k≥0eαeiθ−1kk!xαksin⁡nθdθ =2n!πex/αIm⁡∫0πexp⁡xαeαeiθsin(nθ)dθ,

which is the desired result.

Clearly, the integral representation in (37) boils down to Cesàro's in (36) when *α* = 1 and *x* = 1. Now, applying the explicit formula in (25) gives us the following.

**Corollary 11 .**

The following identity holds:

(41) ∑j≥0jαnj!xαj =2n!πIm⁡∫0πexp⁡xαeαeiθsin(nθ)dθ.

## 5. More Theorems on ${\tilde{D}}_{\left(\alpha \right)}(n;\;x)$

It is known that the *n*th exponential moment of a Poisson random variable *X*, denoted by *E*_*λ*_[*X* ^*n*^], is related to the Bell polynomials *B*_*n*_(*λ*) through the Dobinski's formula. That is,

(42) $$\begin{array}{c} E_{\lambda }\left[X^{n}\right]=B_{n}\left(\lambda \right). \end{array}$$

Also, the *n*th factorial moment of *X* with mean *λ*, denoted by *E*_*λ*_[(*X*)_*n*_], is given by

(43) $$\begin{array}{c} E_{\lambda }\left[{\left(X\right)}_{n}\right]=\lambda^{n}. \end{array}$$

R. B. Corcino and C. B. Corcino [10] obtained a generalization of (42) using the (*r*, *β*)-Bell polynomials as

(44) $$\begin{array}{c} E_{\lambda /\beta }\left[{\left(\beta X+r\right)}^{n}\right]=G_{n,\beta ,r}\left(X\right) \end{array}$$

when *β* = 1 and *r* = 0. We note that identities (42), (43), and (44) can be shown to be particular cases of the generalized factorial moments by Mangontarum and Corcino [14] given by

(45) $$\begin{array}{c} E_{\lambda }\left[{\left(\beta X+\gamma \;|\;\alpha \right)}_{n}\right]=e^{-\lambda }\underset{i\geq 0}{\sum }\frac{{\left(i\beta +\gamma \;|\;\alpha \right)}_{n}}{i!}\lambda^{i},\\ E_{\lambda }\left[{\left(\alpha X-\gamma \;|\;\beta \right)}_{n}\right]=e^{-\lambda }\underset{i\geq 0}{\sum }\frac{{\left(i\alpha -\gamma \;|\;\beta \right)}_{n}}{i!}\lambda^{i}, \end{array}$$

by suitable assignments of the parameters *α*, *β*, *γ*, and *λ*. The following lemma is analogous to (42).

**Lemma 12 .**

The following identity holds:

(46) $$\begin{array}{c} E_{\lambda }\left[{\left(\alpha X\right)}^{n}\right]=\frac{1}{e^{\lambda }}\underset{j\geq 0}{\sum }\frac{{\left(\alpha j\right)}^{n}}{j!}\lambda^{j}, \end{array}$$

where *X* is a Poisson random variable with mean *λ*.

**Proof**

From (8),

(47) $$\begin{array}{c} {\left(\alpha X\right)}^{n}=\overset{n}{\underset{k=0}{\sum }}{\tilde{W}}_{\left(\alpha \right)}\left(n,k\right)\alpha^{k}{\left(X\right)}_{k}. \end{array}$$

Hence by (43),

(48) EλαXn=Eλ∑k=0nW~(α)(n,k)αkXk=∑k=0nW~(α)(n,k)αkEλXk=∑k=0nW~(α)(n,k)αkλk.

Using the explicit formula in (10) and simplifiying further completes the proof.

If the mean of the Poisson random variable *X* is *λ*/*α*, then we have

(49) $$\begin{array}{c} E_{\lambda /\alpha }\left[{\left(\alpha X\right)}^{n}\right]={\tilde{D}}_{\left(\alpha \right)}\left(n;\lambda \right). \end{array}$$

Now,

(50) D~αn;λ=Eλ/α−1+αX+1n=∑k=0nnk−1n−kEλ/ααX+1k=∑k=0nnk−1n−kGk,α,1(λ).

Using the explicit formula of the (*r*, *β*)-Bell polynomials [10]

(51) $$\begin{array}{c} G_{n,\beta ,r}\left(x\right)={\left(\frac{1}{e}\right)}^{x/\beta }\underset{k\geq 0}{\sum }\frac{{\left(x/\beta \right)}^{k}}{k!}{\left(\beta k+r\right)}^{n} \end{array}$$

yields

(52) $$\begin{array}{c} {\tilde{D}}_{\left(\alpha \right)}\left(n;\lambda \right)=\overset{n}{\underset{k=0}{\sum }}\left(\begin{array}{c} n\\ k \end{array}\right){\left(-1\right)}^{n-k}{\left(\frac{1}{e}\right)}^{\lambda /\alpha }\underset{j\geq 0}{\sum }\frac{{\left(\lambda /\alpha \right)}^{j}}{j!}{\left(\alpha j+1\right)}^{k}. \end{array}$$

Hence, we have the following.

**Theorem 13 .**

The following identities hold:

(53) $$\begin{array}{c} {\tilde{D}}_{\left(\alpha \right)}\left(n;\lambda \right)=\overset{n}{\underset{k=0}{\sum }}\left(\begin{array}{c} n\\ k \end{array}\right){\left(-1\right)}^{n-k}{\left(\frac{1}{e}\right)}^{\lambda /\alpha }\underset{j\geq 0}{\sum }\frac{{\left(\alpha j+1\right)}^{k}}{\alpha^{j}j!}\lambda^{j};\\ {\tilde{D}}_{\left(\alpha \right)}\left(n;1\right)=\overset{n}{\underset{k=0}{\sum }}\left(\begin{array}{c} n\\ k \end{array}\right){\left(-1\right)}^{n-k}D_{\alpha }\left(k\right), \end{array}$$

where *D*_*α*_(*k*) is the classical Dowling numbers.

The next theorem is easily deduced from (22) and (46).

**Theorem 14 .**

The following exponential generating functions hold:

(54) $$\begin{array}{c} \underset{n\geq 0}{\sum }E_{\lambda }\left[{\left(\alpha X\right)}^{n}\right]\frac{z^{n}}{n!}=e^{(e^{\alpha z}-1)\lambda };\\ \underset{n\geq 0}{\sum }E_{\lambda /\alpha }\left[{\left(\alpha X\right)}^{n}\right]\frac{z^{n}}{n!}=\underset{n\geq 0}{\sum }{\tilde{D}}_{\left(\alpha \right)}\left(n;x\right)\frac{z^{n}}{n!};\\ \underset{n\geq 0}{\sum }E_{1/\alpha }\left[{\left(\alpha x\right)}^{n}\right]\frac{z^{n}}{n!}=\underset{n\geq 0}{\sum }{\tilde{D}}_{\left(\alpha \right)}\frac{z^{n}}{n!}. \end{array}$$

## 6. The Hankel Transform of ${\tilde{D}}_{\left(\alpha \right)}(n)$

The* Hankel matrix* is a matrix whose entries are symmetric with respect to the main diagonal of the matrix. It had been previously studied by some mathematicians as well as its connections in some areas of mathematics, physics, and computer science. Among these mathematicians were de Sainte-Catherine and Viennot [15], Garcia-Armas and Sethuraman [16], Tamm [17], and Vein and Dale [18]. Further theories and applications of this matrix had been established including the* Hankel determinant* and* Hankel transform*. The determinant of the Hankel matrix is called Hankel determinant, while the sequence of Hankel determinants is called Hankel transform as defined by Aigner [19].

The Hankel determinants had been previously studied by some mathematicians, for instance, Radoux [20] and Ehrenborg [21]. On the other hand, the Hankel transform was first introduced in Sloane's sequence *A*055878 [22] and was first studied by Layman [23]. Aigner [19] established the Hankel transform of the classical Bell numbers. A similar identity was obtained by Mező [11] for the Hankel transform of the *r*-Bell numbers. In a recent paper, Corcino et al. [24] established the Hankel transform of the noncentral Bell numbers which is identical to that of the Bell and *r*-Bell case. A more general case of Hankel transform can also be seen in [24], namely, the Hankel transform of the (*r*, *β*)-Bell numbers. In this section, we are going to establish the Hankel transform of the Translated Dowling Numbers by using Aigner's method.

Let Λ = (*a*_*m*,*k*_) be the infinite lower triangular matrix defined recursively by

(55) $$\begin{array}{c} a_{m,k}=a_{m-1,k-1}+\left(\alpha k+1\right)a_{m-1,k}+\alpha \left(k+1\right)a_{m-1,k+1}, \end{array}$$

where *m* ≥ 1, *a*_0,0_ = 1, *a*_0,*k*_ = 0 if *k* > 0, and *a*_*m*,*k*_ = 0 if *m* < *k*.

Using the reccurence relation in (55), we obtain

(56) ∑m=0∞am,kzm−1(m−1)! =∑m=0∞am−1,k−1zm−1m−1!+αk+1  ×∑m=0∞am−1,kzm−1(m−1)!  +α(k+1)∑m=0∞am−1,k+1zm−1(m−1)!.

This implies that

(57) Ωk′z=Ωk−1(z)+(αk+1)Ωk(z) +α(k+1)Ωk+1(z).

With

(58) $$\begin{array}{c} \Omega_{k}\left(z\right)=e^{\left((e^{\alpha z}-1)/\alpha \right)}\frac{{\left(e^{\alpha z}-1\right)}^{k}}{\alpha^{k}k!}, \end{array}$$

the right-hand side of (57) yields

(59) Ωk−1(z)+(αk+1)Ωk(z)+α(k+1)Ωk+1(z) =e(eαz−1)/αeαz−1k−1αk−1k−1!  +(αk+1)e(eαz−1)/αeαz−1kαkk!  +α(k+1)e(eαz−1)/αeαz−1k+1αk+1(k+1)! =e(eαz−1)/αeαz−1k−1αk−1k−1!  +e(eαz−1)/αeαz−1k−1(eαz−1)αk−1k−1!  +e(eαz−1)/αeαz−1kαkk!  +e(eαz−1)/αeαz−1k(eαz−1)αkk! =e(eαz−1)/αeαz−1k−1αk−1k−1!  +e(eαz−1)/αeαz−1k−1eαzαk−1(k−1)!  −e(eαz−1)/αeαz−1k−1αk−1k−1!  +e(eαz−1)/αeαz−1kαkk!+e(eαz−1)/αeαz−1keαzαkk!  −e(eαz−1)/αeαz−1kαkk! =e(eαz−1)/α+αzeαz−1k−1αk−1k−1!  +e(eαz−1)/α+αzeαz−1kαkk!.

While the left hand side of (57) yields

(60) Ωk′z=e(eαz−1)/αeαz−1k−1αkk!kαeαz +e(eαz−1)/αeαz−1kαkk!1ααeαz=e(eαz−1)/α+αzeαz−1k−1αk−1k−1! +e(eαz−1)/α+αzeαz−1kαkk!.

This implies that the function

(61) $$\begin{array}{c} e^{\left((e^{\alpha z}-1)/\alpha \right)}\frac{{\left(e^{\alpha z}-1\right)}^{k}}{\alpha^{k}k!}, \end{array}$$

where *k* ≥ 0, is a unique solution to the differential equation in (57). Hence, the exponential generating function of the *k*th column of Λ is given by

(62) $$\begin{array}{c} \Omega_{k}\left(z\right)=e^{\left((e^{\alpha z}-1)/\alpha \right)}\frac{{\left(e^{\alpha z}-1\right)}^{k}}{\alpha^{k}k!}. \end{array}$$

Hence, we have the following.

**Lemma 15 .**

Let *Ω*_*k*_(*z*) be the exponential generating function of the *kth* column of matrix Λ. That is,

(63) $$\begin{array}{c} \Omega_{k}\left(z\right)=\overset{\infty }{\underset{m=0}{\sum }}a_{m,k}\frac{z^{m}}{m!}. \end{array}$$

Then

(64) $$\begin{array}{c} \Omega_{k}\left(z\right)=e^{\left((e^{\alpha z}-1)/\alpha \right)}\cdot \frac{{\left(e^{\alpha z}-1\right)}^{k}}{\alpha^{k}k!}, \end{array}$$

where *k* ≥ 0 and $\begin{array}{c} \Omega_{0}(z)=\sum_{m=0}^{\infty }{\tilde{D}}_{\left(\alpha \right)}\left(m\right)\left(z^{m}/m!\right). \end{array}$ That is, the 0-column entries of Λ are the numbers ${\tilde{D}}_{\left(\alpha \right)}(m)$, *m* = 0,1, 2,3….

**Remark 16 .**

When *k* = 0 in (64), we have

(65) $$\begin{array}{c} \Omega_{0}\left(z\right)=\exp\left\{\frac{e^{\alpha z}-1}{\alpha }\right\} \end{array}$$

which is the exponential generating function in (23).

The next lemma is useful in establishing an identity for some matrices whose entries are ${\tilde{D}}_{\left(\alpha \right)}(m)$.

**Lemma 17 .**

Let *s*_*m*_ be the *mth* row of Λ = (*a*_*m*,*k*_). Define

(66) $$\begin{array}{c} s_{m}\circ s_{n}=\underset{k\geq 0}{\sum }a_{m,k}a_{n,k}\alpha^{k}k!. \end{array}$$

Then

(67) $$\begin{array}{c} s_{m}\circ s_{n}=a_{m+n,0}={\tilde{D}}_{\left(\alpha \right)}\left(m+n\right), \end{array}$$

for all nonnegative integers *m* and *n*.

**Proof**

By induction of *m*, if *m* = 0 we have

(68) $$\begin{array}{c} s_{0}\circ s_{n}=\underset{k\geq 0}{\sum }a_{0,k}a_{n,k}\alpha^{k}k!. \end{array}$$

Since *a*_0,*k*_ = 0  ∀*k* > 0,

(69) $$\begin{array}{c} s_{0}\circ s_{n}=a_{0,0}a_{n,0}\alpha^{0}0!=a_{0+n,0}\;\forall n\geq 0. \end{array}$$

Suppose that *s*_*l*_∘*s*_*n*_ = *a*_*l*+*n*,0_ holds for *l* ≤ *m* − 1 and all *n*. Then by (55)

(70) sm∘sn=∑k≥0am,kan,kαkk!=∑k≥0am−1,k−1+αk+1am−1,kiiiiiiiii+α(k+1)am−1,k+1an,kαkk!=∑k≥0am−1,k−1an,kαkk!+∑k≥0αk+1am−1,kan,kαkk! +∑k≥0α(k+1)am−1,k+1an,kαkk!.

Reindexing the summation yields

(71) sm∘sn=∑k≥−1am−1,kan,k+1αk+1(k+1)! +∑k≥0(αk+1)am−1,kan,kαkk! +∑k≥1αkam−1,kan,k−1αk−1k−1!=∑k≥0am−1,kan,k+1αk+1k+1! +∑k≥0αk+1am−1,kan,kαkk! +∑k≥0am−1,kan,k−1αkk!=∑k≥0an,k−1+αk+1an,kiiiiiiiii+αk+1an,k+1am−1,kαkk!.

By (55),

(72) $$\begin{array}{c} s_{m}\circ s_{n}=\underset{k\geq 0}{\sum }a_{n+1,k}a_{m-1,k}\alpha^{k}k!. \end{array}$$

From the inductive hypothesis,

(73) $$\begin{array}{c} s_{m}\circ s_{n}=a_{(n+1)+(m-1),0}=a_{n+m,0}={\tilde{D}}_{\left(\alpha \right)}\left(m+n\right), \end{array}$$

which is pricisely (67).

We are now ready to state the following Hankel transform of the translated Dowling numbers.

**Theorem 18 .**

The numbers ${\tilde{D}}_{\left(\alpha \right)}(m)$ have the Hankel Transform

(74) D~(α)(0)D~(α)(1)D~(α)(2)⋯D~(α)(m)D~(α)(1)D~(α)(2)D~(α)(3)⋯D~(α)(m+1)⋮⋮⋮⋯⋮D~(α)(m)D~(α)(m+1)D~(α)(m+2)⋯D~(α)(2m) =∏r=0mαrr!=αm+12r!!.

**Proof**

Let Λ_*m*_ be the lower triangular submatrix of Λ consisting of the rows and columns numbered 0 to *m*. Then Λ_*m*_ is a matrix with diagonal 1. It follows that det⁡Λ_*m*_ = 1. This implies the determinant of the transpose of Λ_*m*_ is one; that is, det⁡Λ_*m*_^*T*^ = 1. Let ${\hat{\Lambda }}_{m}={\left(\alpha^{r}r!a_{i,r}\right)}_{0\leq i,r\leq m}$. Then

(75) $$\begin{array}{c} \det{\hat{\Lambda }}_{m}=\overset{m}{\underset{r=0}{\prod }}\alpha^{r}r!. \end{array}$$

By (67),

(76) $$\begin{array}{c} {\hat{\Lambda }}_{m}\cdot \Lambda_{m}^{T}={\left(b_{i,r}\right)}_{0\leq i,r\leq m}, \end{array}$$

where $b_{i,r}=\sum_{k=0}^{m}a_{i,k}a_{r,k}\alpha^{r}r!=a_{i+r,0}={\tilde{D}}_{\left(\alpha \right)}(i+r)$. That is,

(77) $$\begin{array}{c} {\hat{\Lambda }}_{m}\cdot \Lambda_{m}^{T}={\left({\tilde{D}}_{\left(\alpha \right)}\left(i+r\right)\right)}_{0\leq i,r\leq m}. \end{array}$$

Thus,

(78) det⁡Λ^m·ΛmT=(det⁡Λ^m)(det⁡ΛmT)=∏r=0mαrr!.

This is the desired result.

**Remark 19 .**

Note that when *α* = 1, we recover from (74) the Hankel transform of the classical Bell numbers of Aigner [19], the Hankel transform of the *r*-Bell numbers of Mező [11], and the Hankel transform of the noncentral Bell numbers in [24]. This makes (74) a generalization of the previously mentioned Hankel transforms. Also, the Hankel transform of the (*r*, *β*)-Bell numbers [24] appears to be analogous to the Hankel transform of the translated Dowling numbers in (74).

Much is yet to be learnt regarding the translated Dowling polynomials and numbers. It is interesting to establish more properties for these polynomials and numbers parallel to the properties of the Bell polynomials and numbers, and their generalizations. The authors also recommend further study regarding the translated *r*-Whitney numbers [3]. The results in this paper might be extended to translate *r*-Dowling polynomials and numbers using the translated *r*-Whitney numbers of the second kind. Another interesting topic can be found in [25] where Corcino et al. obtained the asymptotic formulas for the *r*-Whitney numbers of the second kind as well as the range of validity of each formula. It would be compelling to do the same to the translated Whitney numbers of the second kind.
